# Medication‐Related Information on Social Media: Exploring Public Trust, Perceptions of Credibility, and Influence on Behavior

**DOI:** 10.1002/hsr2.73192

**Published:** 2026-09-04

**Authors:** Abrar H. Abdullah, Lobna Gharaibeh, Fahmi Y. Al‐Ashwal, Rana K. Abu Farha

**Affiliations:** ^1^ Clinical Pharmacy and Therapeutics Department, Faculty of Pharmacy Applied Science Private University Amman Jordan; ^2^ Department of Pharmacy, College of Pharmacy Amman Arab University Amman Jordan; ^3^ Department of Clinical Pharmacy and Pharmacy Practice, Faculty of Pharmacy University of Science and Technology Sana'a Yemen; ^4^ Department of Clinical Pharmacy College of Pharmacy, Al‐Ayen Iraqi University Thi‐Qar Iraq

**Keywords:** health literacy, medication information, misinformation, social media, trust

## Abstract

**Background and Aim:**

Social media has evolved into a major source of health and medical information. This study investigated exposure to medication‐related content on social media among adults in Jordan and examined factors influencing trust, confidence in distinguishing accurate from inaccurate information, and health‐related decision‐making.

**Methods:**

A cross‐sectional online survey was conducted in February 2026 among adults aged ≥ 18 years using convenience and snowball sampling. The questionnaire assessed demographics, social media use, exposure to medication‐related content, trust, verification behaviors, and digital health literacy (eHEALS). Data were analyzed using descriptive statistics and binary logistic regression, with significance set at *p* < 0.05.

**Results:**

A total of 501 participants were included (median age 26, 309 females, 61.7%). Instagram (*n* = 416, 83%), Facebook (*n* = 383, 76.4%) and YouTube (*n* = 376, 75%) were the most common social platforms used. Respondents had frequent encounters with medication information (*n* = 199, 39.7% more than once a day). Content consisted mostly of herbal remedies (*n* = 404, 80.6%), advice from healthcare professionals (*n* = 330, 65.9%) and influencers (*n* = 329, 65.7%). Trust levels were neutral (*n* = 244, 48.7%) to low (*n* = 200, 39.9%). Trust was affected by the nature of the content source (*n* = 426, 85%), nature of the information source (*n* = 426, 85%) and social media platform used (*n* = 309, 61.7%). Most participants (*n* = 309, 61.7%) had encountered false information including false health claims/benefits (*n* = 263, 52.5%) and misleading ads/promotion (*n* = 243, 48.5%). Verification behavior was low to moderate (*n* = 209, 41.7%), with more respondents (*n* = 194, 38.7%) taking action on content seen. Higher education predicted high trust (AOR = 3.05, *p* = 0.009) and low confidence to differentiate reliable information from misinformation predicted high trust (AOR = 3.64, *p* = 0.001). Higher digital health literacy predicted more confidence to differentiate reliable from unreliable information (AOR = 1.082, *p* < 0.001) and low risk to act upon social media advice (AOR = 0.956, *p* = 0.007).

**Conclusion:**

Social media is widely used for medication information, yet trust and digital health literacy remain limited. Exposure to misinformation is common and affects decision‐making. Enhancing digital health literacy, encouraging verification, and promoting credible health communication are critical.

## 1 Introduction

Social media has evolved into a major source of health and medical information, transforming how individuals access, share, and evaluate content. Platforms such as Facebook, YouTube, and Twitter/X enable rapid dissemination of information, improving accessibility and personalization [1]. However, this increased availability also raises concerns regarding content quality, misinformation, and risks to personal privacy [2]. With billions of users worldwide, healthcare professionals increasingly use these platforms for education and knowledge exchange, although safeguarding patient confidentiality remains essential [3, 4, 5].

During public health crises such as COVID‐19, social media has played a key role in disseminating timely and credible information from official sources [6, 7]. Patients, including those with chronic conditions such as cancer, also use social media for information, emotional support, and social interaction [8, 9]. Despite these benefits, misinformation spreads rapidly on these platforms and can outpace accurate information, influencing health behaviors [10, 11].

A low level of digital health literacy, in combination with misleading or automated content, facilitates the proliferation of incorrect health information [12, 13]. Medication related content further increases risks by offering instructions about false dosages, interaction between drugs and use of treatments without evidence [14]. Additionally, product endorsement by social media influencers may reduce awareness of associated dangers of these treatments and thereby lead to manipulation of users [15], thereby leading authorities to issue warnings concerning financial losses, medical treatment delays and other health related adverse consequences [16].

Although research on social media as a source of health and medication‐related information has increased, few studies have examined exposure, information‐seeking behavior, perceived credibility and trust, misinformation identification, e‐health literacy, and medication‐related decision‐making within an integrated framework. Evidence from the Middle East remains particularly limited. Therefore, this study provides an integrated assessment of these interrelated dimensions among adults in Jordan, extending previous research beyond the assessment of medication‐related social media exposure to identify factors associated with users' trust, confidence in identifying misinformation, and subsequent medication‐related decision‐making.

## 2 Methods

### 2.1 Study Design, Participants, and Data Collection

This is cross‐sectional research that was conducted in Jordan in February 2026. The targeted population for this research were adults who regularly use social media and are 18 years of age or older. In order to recruit participants for this study, convenience and snowball sampling methods were used.

The study questionnaire was posted on Google Forms, providing participants with the option to access the questionnaire online. Recruitment of participants occurred via social media platforms (i.e., WhatsApp, Facebook, Instagram). Prior to completing the questionnaire, participants gave their electronic consent to participate in the study.

### 2.2 Questionnaire Development

The study questionnaire was developed after conducting an exhaustive literature search of existing studies on the use of social media in the area of health communication and how users viewed the reliability of medication‐related digital health information. A draft questionnaire was then developed based on this literature review to collect the required data.

The developed questionnaire was divided into five sections. The first section collected demographic information from participants, including age, gender, education level, family income, residential area, whether they had medical insurance, and whether they held a medical‐related degree (e.g., medicine, pharmacy, nursing). The second section collected data about the way participants were personally using social media by looking at what different types of platforms they were using, including, for example, Instagram, YouTube, and Facebook; how often they saw medication‐related content; and what formats (videos, pictures, posts, advertisements) they were seeing when exposed to medication‐related content on those platforms.

The third section looked at how much trust participants had in the medication‐related content they were seeing on social media. This section looked at different sources of medication‐related content (e.g., from a healthcare professional or an influencer compared to a brand), where it was coming from (different social media platforms), and what visual cues (e.g., verified account/number of likes/number of shares) may have influenced participants' levels of trust in the medication‐related content they saw on social media.

The fourth section examined participants' perceptions about whether or not they could determine whether or not the medication‐related content they were exposed to was accurate and/or credible. This included how confident they are in their ability to differentiate between accurate and inaccurate medication‐related information and how they normally react (e.g., do nothing, research further) to misleading or false medication‐related information that they found on social media. The fifth section collected information on the participants' health literacy using the eHEALS scale, which evaluates an individual's health literacy by assessing their ability to find, evaluate, and use health information from reputable sources on the internet.

To assess the validity of the questionnaire content, three academic pharmacists were asked to perform content validation of the questionnaire to evaluate the relevance, clarity, and alignment of the items with the study objectives. Then, the questionnaire was translated into Arabic using a forward‐backward methodology to ensure cultural relevance for the audience being studied. Finally, a pilot test was conducted with a sample of 15 people in order to assess the clarity, comprehensibility, and feasibility of the questions in the questionnaire. Any recommendations resulting from the pilot phase were incorporated into the questionnaire prior to finalizing the questionnaire. Participants included in pilot testing were not included in the final analysis.

The newly developed items were primarily categorical and were analyzed according to their predefined response categories rather than combined into a psychometric scale; therefore, no overall score or internal consistency coefficient was calculated for these items. Internal consistency was only computed for eHEALS and showed an acceptable value for Cronbach's alpha (0.862). eHEALS consists of eight items where responses were measured using a 5‐point Likert scale (1 = strongly disagree to 5 = strongly agree). Items were summed to establish a total eHEALS sum‐score of 8–40 where an higher value implied greater perceived e‐health literacy.

### 2.3 Sample Size Calculation

Prior to conducting this study, a priori power analysis was performed to determine whether the sample size would provide adequate statistical power for the planned analysis. The a priori Sample Size Calculator developed by Daniel Soper was used to estimate the minimum required sample size for multiple regression analysis. Assuming a medium effect size (f^2^ = 0.15), a significance level of α = 0.05, a statistical power of 0.80, and ten predictors in the regression model, the analysis indicated that the minimum required sample size was approximately 118 participants [17].

### 2.4 Ethical Considerations

The study was conducted according to the ethical standards of the Declaration of Helsinki. In addition, the study obtained IRB approval prior to the commencement of data collection from the Institutional Review Board (IRB) at the Applied Science Private University (Ref. No. 2026‐PHA‐4).

Participants provided their electronic informed consent after being informed about the current study, the procedures involved, risks and benefits of the research as well as their rights to participate in, and to withdraw their consent at any time. In order to maintain confidentiality and ensure the anonymity of the participants and protect their privacy, no personal information were collected about the participants (i.e. Name, address) and the recorded data was protected and was accessed only by the team involved in the research.

### 2.5 Data Analysis

Data analysis was carried out using IBM SPSS version 26. The descriptive statistics included means with standard deviations (SD) or medians with interquartile ranges (IQR) for continuous data, depending on the data distribution. For categorical variables, frequencies and percentages were reported. The normality of the data was tested using the Kolmogorov‐Smirnov test.

To identify factors influencing trust in medication‐related social media content, confidence in recognizing misinformation, and the likelihood of making health decisions based on such content, separate binary logistic regression models were constructed for each outcome. Based on the purposeful selection strategy suggested by Bursac et al. [18], variables with *p*‐values < 0.25 in the univariable analyses were candidates for the multivariable logistic regression models. A threshold of 0.25 was chosen because the standard *p* < 0.05 at the univariable screening stage might screen out variables that are important predictors or confounders in the multivariable stage. Selected predictors were chosen additionally based on the clinical and theoretical relevance with the outcome. Multicollinearity was evaluated using variance inflation factors (VIFs) and tolerance values. Model calibration was assessed using the Hosmer–Lemeshow goodness‐of‐fit test, while the Omnibus Test of Model Coefficients was used to assess the overall significance of each model. Cox and Snell and Nagelkerke were reported to provide estimates of the variance explained by the model. All analyses were run at an α level of 0.05.

## 3 Results

### 3.1 The Demographic Characteristics of the Study Participants

The research involved 501 participants, with a median age of 26 years (IQR = 15.0). Of those who participated, most were female (*n* = 309, 61.7%) and the remainder were male (*n* = 192, 38.3%). Approximately half of the participants were employed (*n* = 230, 45.9%), while a smaller proportion were retired (*n* = 41, 8.2%).

Of all participants, the majority had health insurance (*n* = 337, 67.3%). The educational attainment for more than half of those surveyed was that they had completed a university degree (*n* = 272, 54.3%), whereas less than one‐fifth were still in university (*n* = 94, 18.8%), and approximately 15.0% had completed a postgraduate degree (*n* = 75).

In terms of geography, the overwhelming majority of participants were located in the central region of Jordan (*n* = 357, 71.3%) and lived in urban areas (*n* = 448, 89.4%). For those with a degree related to biomedicine, 37.9% (*n* = 190) had one. Further details are available in Table 1.

**Table 1:** Sociodemographic characteristics of the study participants (*n* = 501).

Parameter	Median (IQR)	*n* (%)
Age (years)	26 (15.0)	
Gender		
o Male		192 (38.3)
o Female		309 (61.7)
Employment status		
o Employed		230 (45.9)
o Unemployed		230 (46.0)
o Retired		41 (8.2)
Do you have health insurance?		
o Yes		337 (67.3)
o No		164 (32.7)
Educational level		
o Not educated		3 (0.6)
o School level		57 (11.4)
o University students		94 (18.8)
o University graduate		272 (54.3)
o Post‐graduate degree		75 (15)
Family income		
o < 400 JD/month		78 (15.6)
o 401–800 JD/month		187 (37.3)
o 801–1200 JD/month		107 (21.4)
o > 1200 JD/month		129 (25.7)
Place of residence		
o Center of Jordan		357 (71.3)
o North of Jordan		93 (18.6)
o South of Jordan		51 (10.2)
Do you have a biomedicine‐related degree?		
o Yes		190 (37.9)
o No		311 (62.1)
Living place		
o Urban		448 (89.4)
o Rural		41 (8.2)
o Bedouins		12 (2.4)

*Note:* 1 JD = 1.4 US dollar.

Abbreviation: IQR, Interquartile range.

### 3.2 Social Media Usage and Exposure to Medication‐related Content

Among the 501 participants, Instagram (*n* = 416, 83%) and Facebook (*n* = 383, 76.4%) were the most frequently used social media platforms, followed by YouTube (*n* = 376, 75%), TikTok (*n* = 110, 22%), and Twitter/X (*n* = 100, 20%). Regarding exposure to medication‐related content, 39.7% of participants (*n* = 199) reported encountering such content multiple times a day, while 30.9% (*n* = 155) saw it a few times a week. Only 10.0% of participants (*n* = 50) encountered content once a day, 17.6% (*n* = 88) rarely, and 1.8% (*n* = 9) reported never encountering it.

Participants reported exposure to a variety of medication‐related content types, with herbal or natural remedy advertisements (*n* = 404, 80.6%), healthcare professional advice (*n* = 330, 65.9%), and influencer endorsements of health products (*n* = 329, 65.7%) being the most common. Other frequently encountered content included pharmaceutical advertisements (*n* = 314, 62.7%), alternative medicine and treatments (*n* = 308, 61.5%), personal experiences with medication (*n* = 297, 59.3%), side effects and risks of medications (*n* = 232, 46.3%), over‐the‐counter product recommendations (*n* = 244, 48.7%), drug discount advertisements (*n* = 222, 44.3%), and drug reviews by users (*n* = 194, 38.7%). More details about the exposure to medication‐related content are presented in Table 2.

**Table 2:** Social media usage and exposure to medication‐related content among participants (*n* = 501).

Parameter	*n* (%)
Which social media platforms do you use regularly?	
o Facebook	383 (76.4)
o Instagram	416 (83)
o YouTube	376 (75)
o Twitter/X	100 (20)
o TikTok	110 (22)
How often do you come across medication‐related content on social media?	
o Multiple times a day	199 (39.7)
o Once a day	50 (10)
o A few times a week	155 (30.9)
o Rarely	88 (17.6)
o Never	9 (1.8)
What types of medication‐related content do you encounter on social media?	
o Pharmaceutical Advertisements	314 (62.7)
o Herbal or Natural Remedy Advertisements	404 (80.6)
o Personal Experiences with Medication	297 (59.3)
o Side Effects and Risks of Medications	232 (46.3)
o Influencer Endorsements of Health Products	329 (65.7)
o Healthcare Professional Advice	330 (65.9)
o Alternative Medicine and Treatments	308 (61.5)
o Drug Reviews by Users	194 (38.7)
o Over‐the‐Counter (OTC) Product Recommendations	244 (48.7)
o Drug Discount Advertisements	222 (44.3)

### 3.3 Trust and Credibility of Medication‐Related Content on Social Media

When asked about trust in medication‐related content on social media (Table 3), nearly half of the participants reported a neutral level of trust (*n* = 244, 48.7%), while 39.9% (*n* = 200) reported low trust. Only 11.4% (*n* = 57) expressed high trust.

**Table 3:** Trust and credibility of medication‐related content on social media among participants (*n* = 501).

Parameter	*n* (%)
How much trust do you place in medication‐related content on social media?	
o High trust	57 (11.4)
o Neutral	244 (48.7)
o Low trust	200 (39.9)
What factors influence your trust in medication‐related content on social media?	
o Source of the content (e.g., healthcare professional, influencer, brand)	426 (85)
o Platform where the content is posted	309 (61.7)
o Number of likes	134 (26.7)
o Number of comments	159 (31.7)
o Number of shares	125 (25)
o Visual appeal of the content (e.g., design, quality)	237 (47.3)
o Verified account status (e.g., blue checkmark)	249 (49.7)
o Disclosure of advertising or sponsorship	232 (46.3)
How confident are you in your ability to distinguish between accurate and inaccurate medication‐related content on social media?	
o Confident	282 (56.3)
o Neutral	151 (30.1)
o Unconfident	68 (13.6)

Regarding factors influencing trust, the most frequently reported factor was the source of the content (e.g., healthcare professional, influencer, or brand) (*n* = 426, 85%), followed by the platform where the content is posted (*n* = 309, 61.7%). Other factors included visual appeal of the content (*n* = 249, 49.7%), verified account status (*n* = 237, 47.3%), disclosure of advertising or sponsorship (*n* = 232, 46.3%), number of comments (*n* = 159, 31.7%), number of likes (*n* = 134, 26.7%), and number of shares (*n* = 125, 25%).

Concerning participants' confidence in distinguishing accurate from inaccurate medication‐related content, 56.3% (*n* = 282) were confident, 30.1% (*n* = 151) were neutral, and 13.6% (*n* = 68) were unconfident.

### 3.4 Verifying and Responding to Medication Information on Social Media

Regarding verification behaviors of medication‐related content on social media (Table 4) 41.7% of participants (*n* = 209) reported that they always verify medication‐related content using other sources, such as healthcare professionals or trusted websites, while 30.5% (*n* = 153) reported doing so sometimes and 14.2% (*n* = 71) often. Smaller proportions reported rarely (9.2%, *n* = 46) or never (4.4%, *n* = 22) verifying such information.

**Table 4:** Verification behaviors and impact of medication‐related social media content on health decisions among participants (*n* = 501).

Parameter	*n* (%)
Do you typically verify medication‐related content with other sources (e.g., healthcare professionals, trusted websites)?	
o Always	209 (41.7)
o Sometimes	153 (30.5)
o Often	71 (14.2)
o Rarely	46 (9.2)
o Never	22 (4.4)
Have you ever encountered medication‐related content that you later found out was inaccurate or misleading?	
o Yes	309 (61.7)
o No	73 (14.6)
o Not sure	119 (23.8)
If yes, what type of inaccurate content did you encounter?#	
o Incorrect dosage instructions	108 (21.6)
o False claims about medication benefits	263 (52.5)
o Misinformation about side effects	187 (37.3)
o Unverified personal testimonials	213 (42.5)
o Misleading advertisements	243 (48.5)
When you encounter inaccurate medication‐related content, what was your reaction?^	
o Ignore it	187 (60.5)
o Report it	20 (6.5)
o Search for more information to verify	84 (27.3)
o Share it to correct misinformation	14 (4.5)
o Missing data	4 (1.3)
Have you ever made a health decision based on medication‐related content from social media (e.g., purchasing a product, trying a new medication)?	
o Yes	194 (38.7)
o No	233 (46.5)
o Not sure	74 (14.8)
How likely are you to follow medication‐related advice seen on social media without consulting a healthcare professional?	
o Very likely	34 (6.8)
o Somewhat likely	107 (21.4)
o Neutral	125 (25.0)
o Somewhat unlikely	140 (27.9)
o Very unlikely	95 (19.0)

*Note:* # Multiple responses are allowed. ^ Percentages calculated out of those encountered inaccurate medication‐related content (*n* = 309).

The majority of people surveyed (*n* = 309, 61.7%) reported coming across false and misleading medication‐related information on social media platforms. Only 73 (14.6%) responded that they had not encountered any false medication‐related information, while 119 (23.8%) were uncertain.

Types of false medication information encountered were predominantly medication benefit claims (*n* = 263, 52.5%), misleading promotional advertisements (*n* = 243, 48.5%), unverified testimonials from individuals (*n* = 213, 42.5%), side effect misinformation (*n* = 187, 37.5%), and incorrect drug dosing instructions (*n* = 108, 21.6%).

The primary response to false medical information was to ignore it (*n* = 187, 60.5%), and almost one in four reported searching for additional medical guidance to verify the claim (*n* = 84, 27.3%). A minimal number of individuals (*n* = 20, 6.5%), or 14 (4.5%), reported or helped to correct false medical information on social media, respectively.

The data relating to health‐related decisions shows that over one‐third of survey respondents (*n* = 194, 38.7%) stated that they had made some type of health‐related decision based on medication information located on social media platforms. In contrast, less than half (*n* = 233, 46.5%) mentioned that they had not made any such decision based on medication‐related information posted on social media; 74 (14.8%) were uncertain if they had made a decision on this basis.

A significant percentage of respondents (*n* = 140, 27.9%) identified that they are unlikely to follow medication‐related advice on social networks without consulting a medical provider. Out of the total number of respondents, 95 (19%) were classified as “very unlikely” to follow relations from social media.

### 3.5 Recommendations for Improving the Quality of Medication‐Related Content on Social Media

**Figure 1:**
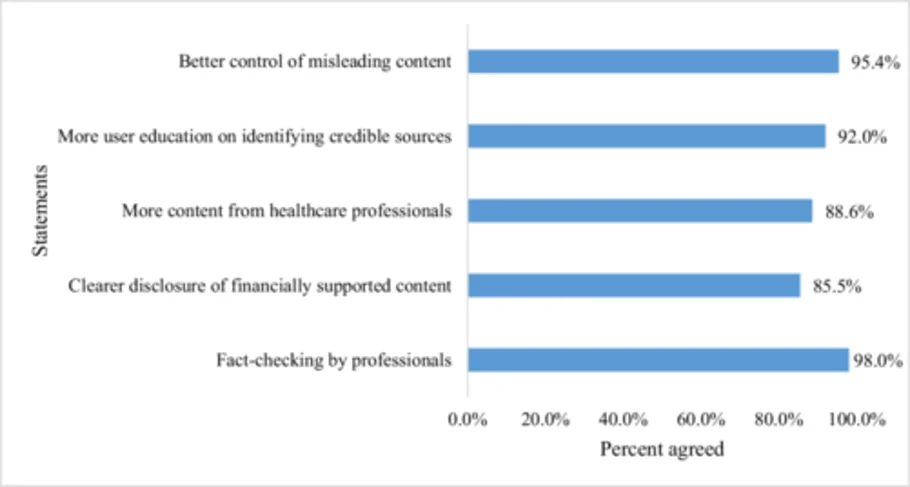
Public Recommendations for Improving the Quality of Medication‐related Content on Social Media (*n* = 501).

The vast majority of participants believed that social media platforms should regulate or moderate medication‐related content (*n* = 462, 92.2%), while 4% (*n* = 20) disagreed and 3.8% (*n* = 19) were unsure. Participants also suggested several measures to improve the quality and credibility of medication‐related content on social media (Figure 1). The most commonly recommended action was fact‐checking by professionals (*n* = 491, 98%), followed by better control of misleading content (*n* = 478, 95.4%) and more user education on identifying credible sources (*n* = 461, 92%). Additionally, 88.6% (*n* = 444) recommended increasing content from healthcare professionals, while 85.8% (*n* = 430) supported clearer disclosure of financially supported or sponsored content.

### 3.6 Digital Health Literacy (eHEALS) Responses among Participants

Participants generally reported low to moderate levels of digital health literacy, with a median total eHEALS score of 17.2 (IQR = 9.1) (Table 5). For the item “I know what health resources are available on the Internet,” the majority disagreed (*n* = 317, 63.3%), 25.9% (*n* = 130) were neutral, and 10.8% (*n* = 54) agreed. Similarly, for “I know where to find helpful health resources on the Internet,” 67.8% (*n* = 340) disagreed, 22.0% (*n* = 110) were neutral, and 10.2% (*n* = 51) agreed. Regarding the next item, “I know how to find helpful health resources on the Internet,” most participants disagreed (*n* = 346, 69.1%), while 25.0% (*n* = 125) were neutral, and 6.0% (*n* = 30) agreed.

**Table 5:** Digital health literacy (eHEALS) responses among participants (*n* = 501).

Statements	Strongly disagree/disagreen (%)	Neutral *n* (%)	Strongly agree/agreen (%)
I know what health resources are available on the Internet.	317 (63.3)	130 (25.9)	54 (10.8)
I know where to find helpful health resources on the Internet.	340 (67.8)	110 (22.0)	51 (10.2)
I know how to find helpful health resources on the Internet.	346 (69.1)	125 (25.0)	30 (6.0)
I know how to use the Internet to answer my health questions.	392 (78.2)	74 (14.8)	35 (7.0)
I know how to use the health information I find on the Internet to help me.	378 (75.4)	86 (17.2)	37 (7.4)
I have the skills I need to evaluate the health resources I find on the Internet.	318 (63.5)	131 (26.1)	52 (10.4)
I can tell high‐quality health resources from low‐quality health resources on the Internet.	323 (64.4)	136 (27.1)	42 (8.4)
I feel confident in using information from the Internet to make health decisions.	218 (43.5)	177 (35.3)	106 (21.2)

For skills in using online health information, 78.2% (*n* = 392) disagreed that they knew how to use the Internet to answer their health questions, and 75.4% (*n* = 378) disagreed that they knew how to use the health information they found to help make health‐related decisions, with smaller proportions reporting neutral (14.8%, *n* = 74% and 17.2%, *n* = 86, respectively) or agreement (7.0%, *n* = 35% and 7.4%, *n* = 37, respectively). Regarding evaluation skills, 63.5% (*n* = 318) disagreed that they had the skills needed to evaluate online health resources, and 64.4% (*n* = 323) disagreed that they could distinguish high‐quality from low‐quality health resources, while 26.1%–27.1% were neutral (*n* = 131 and *n* = 136, respectively) and 8.4%–10.4% agreed (*n* = 42 and *n* = 52, respectively).

Finally, participants expressed slightly higher confidence in using online health information for decision‐making, with 21.2% (*n* = 106) agreeing that they felt confident, 35.3% (*n* = 177) neutral, and 43.5% (*n* = 218) disagreeing.

### 3.7 Factors Associated With Trust in Medication‐Related Content on Social Media

Binary logistic regression was conducted to examine factors associated with high trust in medication‐related content on social media (Table 6). In the adjusted model, educational level and confidence in identifying medication misinformation emerged as significant predictors. Participants with a university‐level education or higher were significantly more likely to report high trust compared to those with school‐level education or below (AOR = 3.05, 95% CI: 1.33–7.04, *p* = 0.009). On the other hand, participants who were neutral or unconfident in distinguishing accurate from inaccurate medication‐related content had higher odds of high trust compared to those who were confident (AOR = 3.64, 95% CI: 1.74–7.59, *p* = 0.001). Other variables, including age, gender, health insurance, family income, having a biomedicine‐related degree, living place, exposure to inaccurate content, and digital health literacy, were not significantly associated with trust in the adjusted analysis.

**Table 6:** Logistic regression analysis of factors associated with trust in medication‐related content on social media (*n* = 501).

Predictors	Dependent variable: trust in medication‐related content on social media[0: neutral/low trust, 1: high trust]					
	COR	95% CI of the COR	*p*‐value#	AOR	95% CI of the AOR	*p*‐value$
Age (years)	1.023	0.998–1.048	0.073^	1.015	0.989–1.041	0.257
Gender						
o Male o Female	Reference 1.522	0.874–2.649	0.138^	1.618	0.863–3.032	0.133
Health insurance						
o Yes o No	Reference 0.888	0.498–1.585	0.688	—	—	—
Educational level						
o School level or below o University level or above	Reference 2.200	1.088–4.447	0.028^	3.054	1.326–7.037	0.009*
Family income						
o < 800 JD/month o > 801 JD/month	Reference 1.363	0.778–2.389	0.279	—	—	—
Biomedicine‐related degree						
o Yes o No	Reference 1.681	0.966–2.925	0.066^	1.894	0.968–3.704	0.062
Living place						
o Urban o Rural/Bedouins	Reference 0.657	0.369–1.170	0.153^	0.741	0.396–1.388	0.349
Encountered inaccurate medication‐related content						
o Yes o No/unsure	Reference 0.772	0.442–1.347	0.362	—	—	—
Digital health literacy (eHEALS total score)	1.079	1.028–1.133	0.002^	1.051	0.997–1.108	0.067
Confidence in identifying medication misinformation in social media content						
o Confident o Neutral/unconfident	Reference 4.180	2.060–8.481	< 0.001^	3.635	1.741–7.590	0.001*

*Note:* # Using simple logistic regression, $ Using multiple logistic regression, ^ Eligible for entry in multiple logistic regression, * Significant at 0.05 significance level.

Abbreviations: AOR, Adjusted Odds Ratio; COR, Crude Odds Ratio.

No evidence of problematic multicollinearity was observed, with VIF values ranging from 1.04 to 1.16 across the predictors. The Omnibus Test showed the model was significant (χ^2^ (7) = 39.28, *p* < 0.001), indicating the predictors effectively distinguished high trust from neutral/low trust. The model explained 7.5% to 14.9% of the variance (Cox and Snell R^2^ = 0.075; Nagelkerke R^2^ = 0.149). The Hosmer–Lemeshow goodness‐of‐fit test indicated good model calibration (χ^2^(8) = 2.649, *p* = 0.954).

### 3.8 Factors Associated With Confidence in Identifying Medication Misinformation

The logistic regression modelling of factors related to the confidence in identifying medication misinformation was significant (χ^2^ (5) = 39.95, *p* < 0.001) (Table 7). Therefore, it was concluded that the predictors included in this model discriminate uniquely between those who were confident and those who were neutral or uncertain in their decisions with moderate ability. No evidence of problematic multicollinearity was observed, with VIF values ranging from 1.03 to 1.14 across the predictors. The variance explained by the comparison of the models produced in this study was minimal at 7.7%–10.3% (Cox and Snell R^2^ to Nagelkerke R^2^, respectively). The Hosmer–Lemeshow goodness‐of‐fit test indicated adequate model calibration (χ^2^(8) = 9.120, *p* = 0.332).

**Table 7:** Logistic regression analysis of factors associated with confidence in identifying medication misinformation (*n* = 501).

Predictors	Dependent variable: confidence in identifying medication misinformation[0: neutral/unconfident, 1: confident]					
	COR	95% CI of the COR	*p*‐value#	AOR	95% CI of the AOR	*p*‐value$
Age (years)	1.008	0.995–1.021	0.208^	1.003	0.989–1.016	0.710
Gender						
o Male o Female	Reference 1.145	0.795–1.647	0.467	—	—	—
Health insurance						
o Yes o No	Reference 1.407	0.967–2.048	0.074^	1.505	1.016–2.228	0.041*
Educational level						
o School level or below o University level or above	Reference 0.644	0.375–1.107	0.111^	0.763	0.426–1.370	0.365
Family income						
o < 800 JD/month o > 801 JD/month	Reference 0.873	0.613–1.244	0.453	—	—	—
Biomedicine‐related degree						
o Yes o No	Reference 1.755	1.210–2.545	0.003^	1.328	0.881–2.002	0.176
Living place						
o Urban o Rural/Bedouins	Reference 0.889	0.601–1.316	0.558	—	—	—
Encountered inaccurate medication‐related content						
o Yes o No/unsure	Reference 1.111	0.773–1.597	0.569	—	—	—
Digital health literacy (eHEALS total score)	1.087	1.055–1.121	< 0.001^	1.082	1.049–1.117	< 0.001*

*Note:* # Using simple logistic regression, $ Using multiple logistic regression, ^ Eligible for entry in multiple logistic regression, * Significant at 0.05 significance level.

Abbreviations: AOR, Adjusted Odds Ratio; COR, Crude Odds Ratio.

The results of the analysis of factors influencing participants' confidence levels in identifying medication information were most strongly correlated with digital health literacy (i.e. eHEALS scores) and insurance status. Participants who were more digitally literate had significantly increased levels of confidence compared to those who were less digitally literate (AOR = 1.082, 95% CI: 1.05–1.12, *p* < 0.001). Conversely, participants without health insurance reported being more confident than those with health insurance (AOR = 1.505, 95% CI: 1.02–2.23, *p* = 0.041). Sociodemographic variables (age, gender, education status, income, biomedical degree, geographic setting, and previous experience with inaccurate content) did not provide significant contributions to confidence.

### 3.9 Factors Associated With Making Health Decisions Based on Medication‐related Social Media Content

The factor analysis using logistic regression found multiple factors significantly associated with making health decisions based upon medication‐related social media content (Table 8). Family income (≥ 801 JD/month) was found to be a significant predictor of making health decisions based on social media at over twice the odds of participants with lower income (AOR = 2.034, 95% CI: 1.379–3.001, *p* < 0.001). Trust in the medication‐related content also emerged as a predictor, where participants with neutral or low trust were statistically less likely to act on social media content than participants having high trust (AOR = 0.352, 95% CI: 0.192–0.645, *p* = 0.001). Notably, participants not encountering inaccurate content (or unsure) had lower odds of making health decisions than participants reporting experiencing inaccurate content (AOR = 0.619, 95% CI: 0.416–0.922, *p* = 0.018). Finally, digital health literacy was inversely associated with making health decisions: participants with lower eHEALS scores had higher odds of acting on social media content (AOR = 0.956, 95% CI: 0.925–0.988, *p* = 0.007). Other factors, including age, gender, educational level, health insurance, biomedicine‐related degree, living place, and confidence in identifying misinformation, were not significant predictors in the adjusted model.

**Table 8:** Logistic regression analysis of factors associated with making health decisions based on medication‐related social media content (*n* = 501).

Predictors	Dependent variable: making health decisions based on medication‐related social media content[0: no/unsure, 1: yes]					
	COR	95% CI of the COR	*p*‐value#	AOR	95% CI of the AOR	*p*‐value$
Age (years)	0.998	0.985–1.011	0.780	—	—	—
Gender						
o Male o Female	Reference 0.910	0.629–1.316	0.617	—	—	—
Health insurance						
o Yes o No	Reference 0.981	0.669–1.436	0.921	—	—	—
Educational level						
o School level or below o University level or above	Reference 1.104	0.632–1.931	0.728	—	—	—
Family income						
o < 800 JD/month o ≥ 801 JD/month	Reference 1.818	1.264–2.614	0.001^	2.034	1.379–3.001	< 0.001*
Biomedicine‐related degree						
o Yes o No	Reference 0.796	0.550–1.151	0.225^	1.085	0.722–1.630	0.694
Living place						
o Urban o Rural/Bedouins	Reference 1.342	0.905–1.988	0.143^	1.418	0.933–2.154	0.102
Trust in medication‐related content						
o High trust o Neutral/Low trust	Reference 0.351	0.199–0.619	< 0.001^	0.352	0.192–0.645	0.001*
Encountered inaccurate medication‐related content						
o Yes o No/unsure	Reference 0.594	0.406–0.867	0.007^	0.619	0.416–0.922	0.018*
Digital health literacy (eHEALS total score)	0.952	0.924–0.980	0.001^	0.956	0.925–0.988	0.007*
Confidence in identifying medication misinformation in social media content						
o Confident o Neutral/unconfident	Reference 0.713	0.495–1.028	0.070^	0.945	0.635–1.408	0.782

*Note:* # Using simple logistic regression, $ Using multiple logistic regression, ^ Eligible for entry in multiple logistic regression, * Significant at 0.05 significance level.

Abbreviations: AOR, Adjusted Odds Ratio; COR, Crude Odds Ratio.

No evidence of problematic multicollinearity was observed, with VIF values ranging from approximately 1.04 to 1.16 across the predictors. The model shows that factors associated with health decision‐making from medication‐related social media content were statistically significant (χ^2^ (7) = 43.79, *p* < 0.001). The model's Cox and Snell R^2^ and Nagelkerke R^2^ values also reveal that they explain between 8.4% and 11.4% of the variance in health decision‐making. The Hosmer–Lemeshow goodness‐of‐fit test indicated adequate model calibration (χ^2^(8) = 8.483, *p* = 0.388).

## 4 Discussion

Most of the participants in the study were females, young, and educated. They were frequently exposed to medication‐related content on social media, mostly herbal or natural products and influencer endorsements. The most frequently used social media platforms were Instagram, Facebook, and YouTube. Our participants were frequent users of medication‐related content on social media, and the trust of consumers was influenced by the source of the content and the platform where the content was posted. Most participants were exposed to false information, but only some reported verification of the information regularly. Low to moderate levels of digital health literacy were reported, and a low rate of skills in digital health literacy. Participants' confidence levels in identifying medication information were most strongly correlated with digital health literacy.

Influencer marketing and promotion of products, including medication, have gained considerable momentum in past years. Gell et al reviewed published research on influencer promotion of prescription drugs. Their findings showed that promotion of prescription drugs by influencers is alarmingly on the rise, which leads to the spread of inaccurate advice among audiences with poor health literacy. This highlights the need for strict regulatory oversight and accountability [19]. This issue is also encountered in over‐the‐counter drugs (OTC). Despite the good knowledge that the public has concerning over‐the‐counter drugs [20], the widespread use of social media and the internet for purchasing and acquiring information was reflected in the OTC market and required organizational adaptation [21].

A small percentage of patients had high trust in the medication‐related content on social media, and only half considered themselves capable of identifying the accuracy of the information. Trust depends mainly on the source of information, where content from doctors or official institutions is perceived as more credible, and the type of platform on which the content was posted. This aligns with previous research emphasizing the importance of source credibility in building trust in health information in social media [22].

Social media is filled with both correct and misleading medical information, which confuses the public on which information should be trusted [23]. Lenca et al discussed the paradox of trust between credible scientific sources and non‐credible sources. While scientific institutions and individuals solve complex problems and rely on science, they are not trusted by the public. Whereas, those with limited consequences for their actions, such as influencers, AI bots, and financially motived people are considered credible [24].

Maximum user engagement through social media renders the spread of misleading medical information to large audiences [25]. Misleading information is particularly disturbing when targeting pregnant women. Van Gelder et al examined 1224 online posts related to medication safety in pregnancy. They reported that most of the information was incorrect and recommended establishing a strong and continuous communication between healthcare providers and pregnant women to combat the utilization of social media for medical information [26].

The type and mode of delivering medical information through social media are diverse. Social media platforms are used by patients to view short videos related to diseases, such as pancreatic or gastric cancer, on health‐related content. However, the quality of these videos is questionable; they have medium quality and low reliability, making them inadequate as a source of information for patients [27, 28].

Despite the fact that more than half of the participants reported encountering false information, they still frequently use social media as a source of information for medication‐related content. Social media platforms possess several advantages, such as accessibility, convenience, and immediacy, making them extremely attractive to consumers. Borges do Nascimento et al investigated the correctness of information posted using mainly YouTube, Twitter, Facebook, and Instagram. They showed that health‐related misinformation ranged from 0.2% to 28.8%, leading to the spread of incorrect medical information, rise in vaccination hesitancy, and delay of seeking adequate healthcare [29].

Half of the participants were confident that they could identify misleading information. In certain instances, individuals with limited knowledge overestimate their own skills, which leads to cognitive bias, a phenomenon known as the Dunning‐Kruger effect. Knowledge overestimation is usually associated with more engagement with social media content [30].

To improve the quality of medication‐related content on social media, participants suggested fact‐checking by professionals, user education, and control and limitation of the misleading content. Other strategies include personalized information campaigns [31, 32] and technology‐based strategies such as fact‐checking websites (“Weibo refutes rumors' and 'Tencent seeks truth”), which can be used to scrutinize the misleading health information [33]. Moreover, the use of machine learning/deep learning models for fact checking to speed up the process was also proposed and examined [34, 35]. Multimedia strategies such as messages from scientific articles, educational credible videos to address misinformation were also investigated [36, 37]. Burke‐Garcia and Hicks suggested the term “Health Communication AI” to combat health misinformation by detecting the authenticity of influential people on social media [38].

Although the highest response concerning eHEALS was confidence in using information from the Internet to make health decisions, participants generally reported low to moderate levels of digital health literacy, which could affect their ability to judge the information posted on social media. Liu et al developed a tool for digital health literacy assessment (DHLA); their findings revealed that a greater DHLA score was associated with a lower risk of health information misjudgment [39]. International evidence similarly indicates that higher digital health literacy is associated with better evaluation of online health information and greater ability to distinguish reliable information from misinformation [39, 40]. Targeted digital health literacy interventions focusing on source evaluation, misinformation recognition, and verification of medication‐related information through credible sources may therefore promote safer information‐seeking and decision‐making behaviors.

Many factors affect trust in medication‐related content on social media. Results indicate that individuals who are not confident in their evaluation skills may be more vulnerable to trusting potentially unreliable information. From a behavioral and cognitive perspective, individuals who are less confident in their ability to evaluate online health information may have greater difficulty distinguishing accurate information from misinformation, which may lead them to place greater trust in medication‐related information encountered on social media. Additionally, those with a higher education level were significantly more likely to report high trust compared to those with lower education levels. Additional factors that exert an impact on the consumers' trust in digital healthcare include the degree of human interaction, perceived risks, privacy concerns, data accuracy, digital literacy, education, and income [41].

Confidence in identifying medication‐related misinformation was most strongly associated with digital health literacy, highlighting the importance of eHealth competencies when individuals search online for health information. Alhewiti study revealed that a higher eHealth literacy level was associated with the ability to distinguish between correct and misleading medical information on social media, leading to trust in specialized health websites and distrust in social media [40]. Consumers who trust the medication‐related content are more likely to make decisions based on social media because they probably can distinguish the misleading information and behave accordingly. Moreover, participants with lower eHEALS scores had higher odds of acting on social media content simply because they cannot critically evaluate information; hence, they are more likely to accept information at face value and act on it. Perceived benefits of social media, frequent use, and exposure to medical content were also associated with a greater influence on decision‐making [42]. The results have important practical implications to various stakeholders. Policymakers need to ensure better surveillance and fact‐checking for health‐ and medication‐related information, while healthcare professionals and pharmacists need to offer trustworthy medication information and help consumers navigate toward trustworthy sources of information. Universities and educational bodies should focus on promoting digital health literacy and debunking fake news, and social media platforms need to reinforce content regulation, source transparency, and reporting features.

This study has several strengths. It focuses specifically on medication‐related content, integrates exposure, trust, behavior, and digital health literacy, and highlights how people manage misinformation. Most importantly, it links literacy to behavior, providing practical insights into how skills influence health decisions. However, the study has several limitations that include the cross‐sectional design, which excludes a causal relationship; therefore, the associations between e‐health literacy, health trust, and health decision‐making should not be viewed as causal and it's directionality can't be identified. Also, using convenience and snowball sampling via social media may also result in selection bias and restricted external validity given the high number of younger, female and more educated participants in the sample so, future conclusions need to be drawn cautiously. The data was self‐reported and is thereby subject to recall bias and social desirability bias (giving acceptable answers). Moreover, the findings are based on perceptions and reported behaviors, which may not accurately reflect actual practices. Additionally, most of the participants were females, young, and educated, which may not be representative of the entire Jordanian population and warrant caution when generalizing the findings.

## 5 Conclusion

This study examined Jordanians' perceptions and behaviors regarding medication information on social media, focusing on trust, credibility, and behavior. The findings confirm that social media is widely used as a source of health information. Despite this widespread use, participants expressed a neutral level of trust in the accuracy of health information published online. Confidence in distinguishing accurate from inaccurate information was moderate, and verification behaviors were modest. Healthcare professionals should actively engage on social media, and fact‐checking systems must be introduced to consumers. Additionally, public awareness campaigns should be conducted to educate the public on the risks of acting on unverified information. Moreover, stronger digital health literacy, clearer source transparency, and proactive engagement by healthcare professionals on social platforms are essential to guarantee maximum benefits of the dissemination of medication‐related information on social media.

## Author Contributions

**Abrar H. Abdullah:** conceptualization, methodology, writing – review and editing, writing – original draft, investigation, visualization, formal analysis, data curation. **Lobna Gharaibeh:** conceptualization, writing – original draft, writing – review and editing, methodology. **Fahmi Y. Al‐Ashwal:** conceptualization, writing – original draft, methodology, writing – review and editing. **Rana K. Abu Farha:** writing – original draft, conceptualization, methodology, writing – review and editing, supervision, formal analysis, visualization.

## Conflicts of Interest

The authors declare no conflicts of interest.

## Author Declaration

All authors have read and approved the final version of the manuscript. The corresponding author, Fahmi Y. Al‐Ashwal, had full access to all of the data in this study and takes complete responsibility for the integrity of the data and the accuracy of the data analysis.

## Transparency Statement

The lead author (Fahmi Y. Al‐Ashwal) affirms that this manuscript is an honest, accurate, and transparent account of the study being reported; that no important aspects of the study have been omitted; and that any discrepancies from the study as planned (and, if relevant, registered) have been explained. The lead author (Abrar H. Abdullah) affirms that this manuscript is an honest, accurate, and transparent account of the study being reported; that no important aspects of the study have been omitted; and that any discrepancies from the study as planned (and, if relevant, registered) have been explained.

## Data Availability

The raw data used to support the findings of this study are available from the corresponding author, Fahmi Y. Al‐Ashwal, upon reasonable request.
